# Factors associated with perinatal substance use among Ethiopian women: an institutional-based cross-sectional study

**DOI:** 10.3389/adar.2023.11913

**Published:** 2023-11-15

**Authors:** Jemal Seid, Emam Mohammed, Yimer Muktar

**Affiliations:** ^1^ Department of Psychiatry, College of Medicine and Health Science, Wollo University, Dessie, Ethiopia; ^2^ Department of Public Health, Kutaber Health Center, Kutaber, Ethiopia; ^3^ School of Veterinary Medicine, Woldia University, Woldia, Ethiopia

**Keywords:** perinatal substance use, pregnancy, women, substance use, Ethiopia

## Abstract

**Introduction:** Substance use during the perinatal period is a significant public health concern, as it can have potential adverse effects on maternal and neonatal health outcomes. Unexpectedly, no previous studies have been conducted to assess the prevalence of substance use during the perinatal period among Ethiopian women. Therefore, this study aimed to determine the magnitude of substance use and its determinant factors during the perinatal period.

**Method:** We conducted a hospital-based descriptive cross-sectional study among a systematically selected sample of 418 women who attended perinatal care between May and July 2022. Data were collected using an interviewer-administered structured questionnaire. Multivariate logistic regression analysis, with a 95% confidence interval and *p*-values less than 0.05, was employed to identify factors associated with substance use behavior.

**Result:** The prevalence of perinatal substance use was found to be 38.3% (95% CI: 33.5–43.5). Of the women who used substances, 109 (26.1%) reported using chat, 46 (11.0%) reported alcohol consumption, and 5 (1.20%) reported using shisha. Factors significantly associated with substance use behavior during the perinatal period included a history of obstetric complications (AOR = 1.722, 95% CI: 1.022–2.902), the presence of chronic medical conditions (AOR = 3.784, 95% CI: 2.164–6.615), experiencing physical abuse (AOR = 5.323, 95% CI: 2.171–13.050), depression (AOR = 1.963, 95% CI: 1.028–3.749), and experiencing sleep disturbances (AOR = 2.016, 95% CI: 0.975–4.168). Conversely, giving birth to a live baby was found to be a protective factor against substance use behavior (AOR = 0.389, 95% CI: 0.187–0.810).

**Discussion:** This study highlights a high prevalence of substance abuse among women during the perinatal period. In light of these findings, a comprehensive approach is recommended to address perinatal substance use among Ethiopian women. This should include the integration of preventive educational programs into perinatal care.

## Introduction

Substance use during the perinatal period poses a significant public health concern due to the potential for adverse maternal and neonatal health outcomes [1]. Globally, more than 190 million people use substances, with alcohol and khat being the most commonly used [2]. Substance use can have detrimental physical, psychological, and social consequences for women, their children, and their families [1, 3, 4]. However, the nature and severity of these effects depend on factors such as dosage, frequency, route of administration, and type of substance [2, 5]. Due to various reasons, women of childbearing age may be more vulnerable to substance use and related disorders [6].

Studies in Europe have reported varying prevalence rates of substance use among women during the perinatal period, ranging from 2% to 27% [5]. The adverse health consequences of substance use affect not only the mother but also the developing fetus and the newborn child [7–13]. Maternal substance use poses a significant risk to child care and disrupts the stability of family life [4, 14]. Common substances used during the perinatal period include alcohol, tobacco, cannabis, and opioids [6].

Substance use during the perinatal period has wide-ranging biological, psychological, social, and economic impacts [10–12]. Maternal substance use, particularly alcohol, is associated with a substantial risk of fetal alcohol spectrum disorders and fetal alcohol syndrome [10]. The World Health Organization (WHO) has identified perinatal substance use, especially alcohol, as a determinant of intellectual and developmental disability [15, 16].

Substance use during the perinatal period is linked to adverse outcomes such as miscarriage, intrauterine growth restriction, early labor, and intrauterine fetal demise [16]. It is strongly associated with reduced birth weight [13, 17], inadequate parenting [14], stillbirth, spontaneous abortion, premature birth, and intrauterine growth retardation [1, 10, 17, 18]. Cigarette smoking and prenatal alcohol exposure are the leading preventable causes of pregnancy-related morbidity, mortality, and developmental problems [16, 19].

Maternal substance use during pregnancy and the postnatal period commonly leads to neurocognitive deficits in children, affecting language, motor, and cognitive functions [17, 20]. Additionally, it can impact other organ systems, such as the heart, skeleton, kidneys, eyes, and auditory system [11, 17, 18]. Substance use is also consistently associated with other mental health conditions, particularly anxiety and depression [21].

A study in the United States reported that the prevalence of substance use in the prenatal period was 25.8% and that unemployment, unmarried status, and psychopathology played a role in it [21]. A study in the United Kingdom also reported that alcohol consumption varied widely (from 10% to 54%) [22]. A study conducted at a university in Ethiopia shows that 46.2% of the study population had used at least one substance in their lifetime and that gender, religion, and income were significantly associated factors [2]. Another study conducted in Jimma town among pregnant women reported that 37.9% were current substance users and that educational status, family history of substance use, occupational status, and gestational age were all found to be associated with substance use [3]. To our knowledge, there are no published Ethiopian data on substance use among women in the perinatal period. Therefore, the aim of this study is to assess the extent of substance use and associated factors among women attending perinatal services at the Kutaber district government health facilities and at the Boru Meda General Hospital in the Amhara region of Ethiopia.

## Methods and materials

A cross-sectional study was conducted in Kutaber District and at the Boru Meda General Hospital, Amhara, Ethiopia, from March to June 2022. The study included women who received perinatal services during this period. Eligible participants were women aged 18 years or older, with regular antenatal and postnatal care attendance, and those willing to participate. Women with hearing or speech impairments and those who were severely ill and unable to complete the questionnaires were excluded. The desired sample size of 418 was calculated using a double population proportion formula with a 95% confidence level, 80% study power, a 2:1 ratio of exposed to unexposed groups, an outcome rate of 37.8% in the unexposed group, and an adjusted odds ratio of 1.71 from a previous study [23].

We recruited the study participants using a systematic random sampling method. The total estimated number of women who visited the study area per day is 65 (30 and 35 in the Kutaber district health facility and at the Boru Meda General Hospital, respectively). In 1 month, a total of 1,430 women (663 in Kutaber and 767 at the Boru Meda General Hospital) were visited. Since the number of required study participants is 418, the sampling interval (k) was calculated as 1430/418 = 3. Accordingly, we selected one participant for every three women. A proportional allocation was made to the study area. These 194 and 224 women were recruited from the Kutaber district health facility and the Boru Meda General Hospital, respectively. Finally, the first starting number of each study site was picked randomly using the lottery method from the registration counter. We used a structured interviewer-administered questionnaire to obtain socio-demographic, obstetric, behavioral, and other relevant associated information from the respondents.

The data collection tool was a structured questionnaire consisting of socio-demographic, obstetric substance use-related characteristics (substance use before and during the perinatal period), and personal and social factors. The questionnaire was first prepared in English and then translated into Amharic languages and back into English to facilitate understanding and ensure consistency during administration. To screen for perinatal substance use, a modified form of ASSIST was used, which was developed by an international group of substance abuse researchers at WHO to detect and manage substance use and related problems in primary and general medical care settings. It consists of a series of questions asking individuals about the frequency and quantity of substance use in the past 3 months. The average reliability test coefficients for ASSIST ranged from Cronbach’s α of 0.58–0.90, indicating good to excellent reliability [24, 25]. Perinatal depression, anxiety, and stress were assessed by using the Depression Anxiety and Stress Scale-21 (DASS-21), which contains relevant items (seven statements each for depression, anxiety, and stress subscales) to recognize the presence and severity of symptoms of these three psychological conditions. The internal consistency and reliability of the DASS-21 were very impressive in Ethiopia, with Cronbach’s α of 0.75, 0.72, 0.86, and 0.95 for the DASS depression, anxiety, stress, and total scales, respectively [26]. These coefficients demonstrated good internal consistency, with cut-off values for DASS depression, anxiety, and stress of 9, 7, and 14, respectively [26–30].

The Epworth Sleepiness Scale was used to measure a subjective report of daytime sleepiness in women. The ESS has excellent psychometric validity for screening daytime sleepiness in the Ethiopian population [31, 32] with a reliability test of 0.725 [32]. Women with an ESS score >10 were included in the sleepy group, and those with a score ≤10 were included in the non-sleepy group [33–37].

Socio-demographic characteristics and obstetric variables such as pregnancy trimester, previous pregnancy and labor complications, previous history of stillbirth, previous history of abortion, plan of current pregnancy, previous psychiatric history, baby’s father’s support, partner’s feelings on current pregnancy, and community support were all collected by a structured and pre-tested questionnaire.

Data were collected by four BSc graduate female clinical nurses and under the supervision of two MSc public health professionals. The completeness and consistency of the collected data were checked daily by the supervisors and the principal investigator. Ethical approval for the study was granted by the Institutional Review Board (IRB) of Wollo University College of Medicine and Health Science, Ethiopia. A letter of permission was obtained from the Kutaber District Health Office and Boru Meda Hospital. Before starting to fill out the questionnaire, written consent was collected from every participant.

### Data quality control

To ensure data quality, 2 days of training (1 day theoretical and 1 day practical) were given to data collectors and supervisors. A pretest was conducted on 10% of the total sample size attending the perinatal service at Haroye Health Center to assess the simplicity, flow, and consistency of the questionnaire. Data entry was performed using Epi-Info™ version 7.1. After a full inspection for completeness and consistency, these were then exported to the Statistical Package for the Social Sciences, SPSS v. 26 (IBM, Armonk, USA) for analysis. Categorical descriptive results were expressed as an actual number (frequency). To determine the association between independent and dependent variables, binary logistic regression was performed. Variables in bivariable analysis with a *p* < 0.2 were selected for multivariable logistic regression. In the multivariable analysis, variables with a *p* < 0.05 and a 95% CI were considered significant factors for perinatal substance use. Hosmer and Lemshow goodness-of-fit tests were performed to assess model fitness at *p* > 0.05. In addition to *p* values, crude odds ratio and adjusted odds ratios with a 95% CI were reported.

Ethical clearance was obtained from the Institutional Review Board (IRB) of Wollo University College of Medicine and Health Science. A permission letter was obtained from the Kutaber District Health office and Boru Meda Hospital and written consent was taken from each study participant. Personal identifying details were not recorded.

## Results and discussion

In this study, 418 women were recruited, resulting in a 100% response rate. All respondents were between 18 and 45 years of age, with a mean age of 29.9 ± 5.44 (SD). The median age was 29.5 years, and the mode was 30 years. Among the participants, 286 (68.4%) were aged 24–30 years, 242 (57.9%) lived in rural areas, 399 (95%) identified as Amhara by ethnicity, 329 (78.7%) were married, and 280 (67%) identified as Muslim by religion (Table 1).

**TABLE 1:** Sociodemographic characteristics of study participants (*n* = 418).

Variables	Response	Frequency	Percentage
Age in years	<24	79	18.9
	24–35	286	68.4
	>35	53	12.7
Residence	Rural	242	57.9
	Urban	176	42.1
Ethnicity	Amhara	399	95.5
	Tigray	14	3.3
	Afar	5	1.2
Religion	Muslim	280	67.0
	Orthodox	123	29.4
	Catholic	6	1.4
	Protestant	8	1.9
	Others	1	0.2
Educational status	Unable to read and write	108	25.8
	Primary school	156	37.4
	Secondary school	87	20.8
	Diploma or higher	67	16.0
Marital status	Single	53	12.7
	Married	329	78.7
	Divorced	23	5.5
	Widowed	13	3.1
Employment	Student	38	9.1
	Not employed	300	71.8
	Employed	80	19.1
Monthly income in birr	Not sure	244	58.4
	Up to 1,000	37	8.9
	>1,001	137	32.8
Social support	High	124	29.7
	Medium	182	43.5
	Low	112	26.8
Marital conflict	Absent	198	47.4
	Present	218	52.2
	No husband	2	0.4

Concerning their feelings about the current pregnancy/child, 355 women (80.1%) reported feeling happy, 316 (75.6%) stated that the pregnancy/child was planned, 262 (62.7%) did not have excessive pregnancy-related concerns, and 342 (81.8%) indicated that the pregnancy/child was post-marital (Table 2).

**TABLE 2:** Pregnancy and social characteristics of study participants (*n* = 418).

Variable	Response	Frequency	%
Feelings about current pregnancy/child	Unhappy	83	19.9
	Happy	335	80.1
Current pregnancy	Unplanned	102	24.4
	Planned	316	75.6
Excessive concern about pregnancy	Present	156	37.3
	Absent	262	62.7
Premarital pregnancy	No	342	81.8
	Yes	76	18.2
History of obstetric complications	Present	174	41.6
	None	244	58.4
Poor attitude toward current pregnancy	YES	95	22.7
	NO	323	77.3
Number of children	No children	101	24.2
	1–3 children	255	61.0
	4 or more children	62	14.8
Delivery outcome (last birth)	Live birth	75	17.9
	Stillbirth	329	78.7
	Still pregnant	14	3.3
Adverse life events	No	291	69.6
	Yes	127	30.4
Family member illness	Absent	322	77.0
	Present	96	23.0
History of domestic violence?	No	365	87.3
	Yes	53	12.7
History of sexual abuse?	No	358	85.6
	Yes	60	14.4
Financial conflict	Absent	213	51.0
	Present	205	49.0
History of physical abuse?	No	357	85.4
	Yes	61	14.6

Clinically, among the women recruited, 48 (11.5%) had a history of previous psychiatric problems, 43 (10.3%) had attempted suicide, and 108 (25.8%), 163 (39.0%), 72 (17.2%), and 70 (16.7%) experienced symptoms of depression, anxiety, stress, and sleepiness, respectively. Additionally, 132 women (31.6%) had other medical conditions (Table 3).

**TABLE 3:** Clinical characteristics of study participants (*n* = 418).

Variable	Response	Frequency	%
History of psychiatric illness	No	370	88.5
	yes	48	11.5
Family history of substance use	No	281	67%
	Yes	137	33%
History of suicide attempts	No	375	89.7
	Yes	43	10.3
History of depression	No	310	74.2
	Yes	108	25.8
History of anxiety	No	255	61.0
	Yes	163	39.0
Stress levels	No	346	82.8
	Yes	72	17.2
Daytime sleep	Has overslept	348	83.3
	Has not overslept	70	16.7
Chronic medical condition	No	286	68.4
	Yes	132	31.6

Of the 418 women in the sample, 160 (38.3%) reported using substances. Among substance users, 109 (26.1%) used khat, 46 (11.0%) consumed alcohol, and 5 (1.20%) used shisha. Of these substance users, 63 (46.0%) had a family history of substance use. In terms of frequency, 20 (4.8%) used substances daily, 75 (17.9%) weekly, 39 (9.3%) monthly, 9 (2.2%) irregularly, and 17 (4.1%) only once or twice in their lifetime.

### Factors associated with substance use

To identify factors associated with substance use, bivariate analyses were conducted between substance use and various sociodemographic, obstetric, psychosocial, and clinical variables. Factors with *p* values less than 0.20 in bivariate analyses were included in a multivariate logistic regression for further analysis.

Substance use was significantly associated with having a history of obstetric complications (AOR = 1.722, 95% CI: 1.022–2.902), the presence of chronic medical conditions (AOR = 3.784, 95% CI: 2.164, 6.615), physical abuse (AOR = 5.323, 95% CI: 2.171, 13.050), depression (AOR = 1.963, 95% CI: 1.028, 3.749), and sleepiness (AOR = 2.016, 95% CI: 0.975, 4.168). On the other hand, having a live birth was a protective factor against perinatal substance use (AOR = 0.389, 95% CI: 0.187, 0.810) (Table 4).

**TABLE 4:** Factors associated with substance use (bivariate and multivariate logistic regression analysis) (N = 418).

Variables	Substance use		Bivariate analysis			Multivariate analysis		
	No	Yes	*p*-value	COR	95% CI	AOR	95% CI	*p*-value
History of obstetric complications								
No	77	167	1					
Yes	83	91	0.001*	1.978	1.324–2.956	1.722	1.022–2.902	0.041**
Delivery outcome (last birth)								
Live birth	127	202	1					
Stillbirth	30	45	0.823	.943	.565–1.574	0.389	0.187–0.810	0.012**
Still pregnant	3	11	0.197*	.409	.105–1.590			
Do you have any other chronic medical conditions?								
No	72	214	1					
Yes	88	44	0.000*	5.944	3.791–9.320	3.784	2.164–6.615	0.000**
Have you ever been physically abused in the past?								
No	112	245	1					
Yes	48	13	0.000*	8.077	4.207–15.507	5.323	2.171–13.050	0.000**
Depression								
No	94	216	1					
Yes	66	42	0.000*	3.611	2.288–5.700	1.963	1.028–3.749	0.041**
Daytime sleepiness								
No	118	230	1					
Yes	42	28	0.000*	2.924	1.726–4.953	2.016	0.975–4.168	0.038**

Note *: *p*-value <0.20 of bilateral regression, **:- Statistically significant at *p* < 0.05 and *p*-value of Hosmer and Lemeshow goodness-of-fit test was = 0.354.

The study found that the overall prevalence of substance use among women in the perinatal period was 38.3% (95% CI: 33.5–43.45). This prevalence is similar to a systematic meta-analysis study among pregnant women in the Amhara region, which reported a prevalence of 36.7% [38]. The prevalence of substance use in this study was higher than in other studies conducted in the USA among pregnant women at 25.8% [21], in Iran among pregnant women at 15% [39], and in Ethiopia among college students at 26.6% [40]. In contrast, the prevalence in this study is lower than in another study in Ethiopia among university students (55.2%) [2].

The variation could be due to the differences in study participants, geographical location, demographic characteristics, methodological differences, sample size, economic factors, lifestyle, substance availability, early diagnosis and treatment, availability of counseling services, and criteria used to define substance use.

In terms of associated factors, the study found that women with a history of obstetric complications were more likely to engage in risky substance use during the perinatal period (AOR = 1.722, 95%: 1.022–2.902). This is consistent with other studies conducted in Germany [13], in the United States [41], in South Africa [42], and in Ethiopia [10]. This suggests that stress and anxiety related to previous complications may lead some women to turn to substances as a coping mechanism.

Similar to other studies in the United States of America (43, 44), women with chronic medical illnesses were three times more likely to engage in substance use during the perinatal period (AOR = 3.784, 95% CI: 2.164, 6.615). This may be due to the added stress and physical discomfort associated with managing a chronic illness in combination with pregnancy.

The study also shows that women with a history of physical abuse were five times more likely to use perinatal substances (AOR = 5.323, 95%: 2.171, 13.050). This was supported by the study conducted in the USA [45]. The association may be due to the fact that women who experience physical abuse may resort to substance use as a way of coping with the trauma or stress associated with the abuse.

Compared with women without current depression symptoms, women with current depression symptoms were twice as likely to use substances in the perinatal period (AOR = 1.963, 95% CI: 1.028, 3.749). The result was consistent with other studies in the United States [21] and in Ethiopia [10]. The association may be due to the fact that women with poor mental health are more likely to use a substance for self-treatment. So, this highlights the importance of addressing mental health issues during the perinatal period to reduce the risk of substance use.

In agreement with another study conducted in New York [46], there was a trend indicating an association between sleepiness and perinatal substance use. This suggests that sleep disturbance or hypersomnia may be linked to substance abuse as women seek ways to manage their sleep-related problems.

Interestingly, the study found that having a live birth was a protective factor against substance use during the perinatal period. This finding is also in line with other studies of pregnant women in different parts of the world [4, 15, 18, 39]. This may suggest that women who successfully give birth to a healthy child are less inclined to use substances during the perinatal period. Further research is needed to fully understand this protective effect.

## Conclusion

In the current study, a significant proportion of mothers were shown to use substances in the perinatal period in a risky way (for the mother, the growing fetus, and the born child). Having a history of obstetric complications, along with the presence of chronic medical illness, physical abuse, depression, and sleepiness, were found to be significantly associated with substance use behavior in the perinatal period. Conversely, having a live birth was a protective factor for substance use behavior in the perinatal period. In light of these findings, a comprehensive approach is recommended to address perinatal substance use among Ethiopian women. This should include the integration of preventive educational programs into perinatal care. Healthcare providers should be trained to screen women for substance use and associated risk factors, such as a history of obstetric complications, chronic illness, physical abuse, and depression, and to provide appropriate counseling and connect women to support services for substance use and mental health issues. A multidisciplinary approach, involving healthcare professionals, social workers, and counselors, is essential to effectively tackle this public health challenge and improve the wellbeing of both mothers and newborns during the perinatal period.

### Recommendation

Implement routine screening for substance use and associated risk factors as part of perinatal care to identify at-risk women. Promote integrated care that addresses both physical and mental health needs during the perinatal period, especially for women with chronic medical conditions, physical abuse, psychopathology, and a history of obstetric complications. Develop support programs and interventions aimed at reducing substance use during the perinatal period, with a focus on addressing the identified risk factors. Provide health education about the effects of substance use on the mother, fetus, and child for all women in antenatal and postnatal care follow-up units.

## Data Availability

The original contributions presented in the study are included in the article/supplementary material, further inquiries can be directed to the corresponding author.

## Abbreviations

ANC, Antenatal Care; AOR, Adjusted odds ratio; ASSIST, Alcohol, Smoking, and Substance Involvement Screening Test; CI, Confidence Interval; COR, Crude odds ratio; DASS-21, Depression, Anxiety and Stress Scale-21; PNC, Postnatal Care; SPSS, Statistical Package for Social Science; USA, United States of America; WHO, World Health Organization.
